# *OsDWARF10*, transcriptionally repressed by *OsSPL3*, regulates the nutritional metabolism of polished rice

**DOI:** 10.3389/fpls.2023.1322463

**Published:** 2023-12-07

**Authors:** Kang Li, Yan Cheng, Chuanying Fang

**Affiliations:** ^1^ Hainan Yazhou Bay Seed Laboratory, Scool of Breeding and Multiplication (Sanya Institute of Breeding and Multiplication), Hainan University, Sanya, China; ^2^ School of Tropical Agriculture and Forestry, Hainan University, Haikou, China

**Keywords:** strigolactones, polished rice, metabolome, *OsD10*, *OsSPL3*, transcriptional regulation

## Abstract

Strigolactone (SL) plays essential roles in plant development and the metabolism of rice leaves. However, the impact of SL on the accumulation of nutritional metabolites in polished rice, as well as the transcription factors directly involved in SL synthesis, remains elusive. In this study, we performed a metabolome analysis on polished rice samples from mutants of an SL biosynthetic gene, *OsDWARF10* (*OsD10*). Compared with those in the wild type plants, primary and secondary metabolites exhibited a series of alterations in the *d10* mutants. Notably, the *d10* mutants showed a substantial increase in the amino acids and vitamins content. Through a yeast one-hybridization screening assay, we identified OsSPL3 as a transcription factor that binds to the *OsD10* promoter, thereby inhibiting *OsD10* transcription *in vivo* and *in vitro*. Furthermore, we conducted a metabolic profiling analysis in polished rice from plants that overexpressed *OsSPL3* and observed enhanced levels of amino acids and vitamins. This study identified a novel transcriptional repressor of the SL biosynthetic gene and elucidated the regulatory roles of OsSPL3 and *OsD10* on the accumulation of nutritional metabolites in polished rice.

## 1 Introduction

Plant metabolites are critical sources of energy and nutrition for humans (Wurtzel and Kutchan, 2016; Li et al., 2019), providing essential elements like nine essential amino acids (valine, leucine, isoleucine, phenylalanine, tryptophan, threonine, lysine, methionine, and histidine) and nearly all vitamins needed for human metabolic processes (Li et al., 2021b; Shi et al., 2022; Shi et al., 2023). As a prominent staple crops, rice (*Oryza sativa*) sustains over half of the global population’s food requirements (Fitzgerald et al., 2009). However, it cannot meet humans’ daily dietary nutritional needs due to insufficient nutrient levels (Li et al., 2022; Ren et al., 2023). Therefore, there is an imperative to unravel the genetic basis underlying the accumulation of healthy metabolites in polished rice.

The accumulation of plant metabolites is influenced by various intrinsic and extrinsic factors (Jin et al., 2015; Qu et al., 2016; Fang et al., 2019; Qi et al., 2021), with hormones serving integral functions (Murcia et al., 2017; Nguyen et al., 2022). Strigolactone (SL) is a novel class of phytohormones derived from the carotenoid pathway, and starting from the isomerization of all-trans-β-carotene into 9-cis-β-carotene catalyzed by β-carotene isomerase (encoded by *DWARF27* in rice) (Lin et al., 2009). Subsequently, 9-cis-β-carotene is cleaved by carotenoid cleavage dioxygenase 7 (CCD7, encoded by *OsHTD1*/*OsDWARF17* in rice) and CCD8 (encoded by *OsDWARF10/OsD10* in rice) (Arite et al., 2007; Wang et al., 2020), resulting in the formation of carlactone, a key intermediate in SL biosynthesis. Further modifications of carlactone by cytochrome P450 enzymes, 4-deoxyorobanchol synthase, and 4-deoxyorobanchol hydroxylase result in the formation of various types of SL in rice (Abe et al., 2014; Yoneyama et al., 2018; Chen et al., 2023). SL plays a wide range of regulatory roles in the germination of parasitic plants, in the formation of arbuscular mycorrhizae, environmental adaptabilities, and plant growth and development (Gomez-Roldan et al., 2008; Kapulnik et al., 2011; Brewer et al., 2013; Mostofa et al., 2018). In a recent study, we discovered significant metabolic changes in the leaves of two rice SL mutants, *d10* and *d14* (a sensing mutant). The mutants showed an increased accumulation of most lipids and terpenoids compared with ZH11, while the flavonoid pathway was significantly inhibited (Zhou et al., 2020). These suggest that SL controls the metabolic flux distribution in rice leaves. However, the role of SL in regulating the metabolism of nutritional metabolites, especially amino acids and vitamins, in polished rice remains unclear.

Many efforts have been devoted to revealing the regulation of SL biosynthesis. SL synthesis genes *DWARF27*, *CCD7*, and *CCD8* express at higher levels under environmental stress, including phosphorus deficiency, nitrogen deficiency, and sulfur deficiency (Yoneyama et al., 2013; Shindo et al., 2018; Trasoletti et al., 2022). In addition, the accumulation of SL is regulated by various phytohormones. For instance, auxin enhances SL production by promoting the expression of *CCD7* and *CCD8*, while abscisic acid, cytokinin, and gibberellin inhibit the accumulation of SL (Xu et al., 2015; Wang et al., 2018; Tian et al., 2022). Moreover, impaired SL biosynthesis or signaling induces *CCD7* and *CCD8* genes expression and SL accumulation in diverse species, suggesting a negative feedback regulation on SL content (Mashiguchi et al., 2021). However, few transcription factors have been characterized to regulate SL biosynthesis genes directly.

SQUAMOSA promoter-binding-like transcription factors (SPLs) are plant-specific transcription factors that harbor a highly conserved 76-amino acid SBP domain, which enables them primarily to bind DNA sequences with a GTAC core sequence (Wang et al., 2009). The function and underlying molecular mechanism of *SPLs* in regulating plant architecture, inflorescence architecture, panicle architecture, grain size, and plant stress resistance have been extensively studied (Chen et al., 2010; Wang et al., 2015a; Xu et al., 2016; Feng et al., 2023; Li et al., 2023). Notably, SPLs play a crucial regulatory role in hormone signaling pathways. OsSPL14, also known as IPA1, directly activates the transcription of *DWARF53*, an inhibitory factor in the SL signaling (Song et al., 2017). However, whether *SPL* transcription factors directly regulate the synthetic genes of SL remains unknown.

Although significant progress has been made in the study of SL synthetic genes and *OsSPLs* in rice, the relationship between them and their role in the metabolism of polished rice have yet to be fully elucidated. In this study, we conducted a metabolomic analysis of *d10* mutant polished rice. We observed significant changes in the contents of various metabolites, including amino acids, vitamins, lipids, organic acids, sugars, flavonoids, and phenolic acids, compared with ZH11. Most of the amino acids and many B family vitamins showed significant increases. In addition, we discovered that the transcription factor OsSPL3 directly binds to the *OsD10* promoter, acting as a negative regulator of *OsD10* gene expression in rice. Moreover, our analysis of the metabolomic profiles of transgenic plants overexpressing *OsSPL3* revealed its role in promoting the accumulation of numerous amino acids and vitamins in polished rice. These findings suggest that OsSPL3 directly represses *OsD10*, thereby influencing the nutritional metabolism in polished rice.

## 2 Materials and methods

### 2.1 Plant materials and growth conditions

The SL biosynthetic mutants *d10*, which was generated in our previous work (Liu et al., 2020), and its’ corresponding background line Zhonghua11 (*O. sativa L*. japonica, ZH11) were selected as the plant materials for this study. *OsSPL3*-overexpressing plants were generated under control of the maize (Zea mays) ubiquitin promoter in ZH11.The rice plants used in this study were planted in two replicates in paddy fields in Lingshui (Hainan Province, China; longitude 110°110′ E, latitude 18°300′ N). The seeds were germinated on moist filter paper at 37°C for three days before being transferred to seedbeds in mid-June. Subsequently, the seedlings were transplanted to the field in mid-July following standard agricultural practices for field management. At maturity, grains from five plants of each accession were harvested to determine the amino acid content. For the cultivation of rice seedlings, a growth chamber was used under controlled conditions with a photoperiod of 16 h light (28°C) and 8 h dark (26°C). The nutrient solution used for seeding followed the previous protocol (Shi et al., 2023), and the solution was changed every five days.

### 2.2 Gene cloning, vector construction, and transformation

Gateway recombination reactions (Invitrogen, Waltham, MA, USA) were performed to generate the overexpression construct of *OsSPL3*, the full-length coding sequence (CDS) of *OsSPL3* (*indica*, Minghui63, MH63) was amplified and cloned into the vector pJC034. The construct was introduced into Agrobacterium tumefaciens EHA105 and subsequently transferred into ZH11, as described previously (Hiei et al., 1994). All primers used to generate the constructs are listed in **Supplementary** **Table S1**.

### 2.3 Metabolite sample preparation

Mature seeds were harvested at the mature stage in 2021. Five independent plants were harvested and combined into a biological replicate. Each sample contained 15 grains of polished rice for metabolite extraction. The samples were ground into powder using a mix mill MM400, (Retsch, GmbH, Haan, Germany) with a zirconia bead for 1 min at 30 Hz. Then, 100 mg powder was weighed, and 70% methanol aqueous solution was added to achieve a concentration of 0.1 mg mL^−1^. Next, ultrasonication was used to extract the sample mixture at 40 Hz for 30 min. The mixture was then centrifuged and filtered (SCAA-104, 0.22 mm pore size; ANPEL, Shanghai, China, http://www.anpel.com.cn/).

### 2.4 Metabolite profiling

The quantification of metabolites was carried out in the multiple reaction monitoring (MRM) mode using LC-MS 8060 (Shimadzu, Kyoto, Japan). The analytical conditions were as described previously (Chen et al., 2013; Wu et al., 2023). The detection of material metabolites, retention time, mass-to-charge ratio, and MS/MS2 of all detectable ions was recorded. The ion characteristics of the sample were automatically matched with the internally established reference libraries of chemical standard entries to identify metabolites.

### 2.5 Yeast-one-hybrid assays

The Matchmaker Gold Yeast One-Hybrid System (Clontech, Mountain View, CA, USA) was used for yeast one-hybridization (Y1H) screening. According to the manufacturer’s protocol, a 500 bp length DNA fragment upstream located 301 bp to 800 bp upstream of *OsD10* initiation codon was cloned into pHis2 yeast vector (Clontech, Mountain View, CA, USA) as a bait. The bait-reporter yeast strain was then transformed with the pGADT7-based rice cDNA library generated from the roots and leaves of rice. Transformants were spread on medium minus Trp, Leu, and His with 20 mM 3-amino-1,2,4-triazole (3-AT; Sigma-Aldrich (Shanghai), Shanghai, China). Then colonies were picked for plasmid extraction and sequenced.

The promoter region (-580 to -628 upstream of the ATG codon) of *OsD10* was inserted into the pHis2 yeast vector, a bait reporter. The coding sequence of *OsSPL3* was cloned into the pGADT7 vector. pGADT7-*OsSPL3* and pHis2-*OsD10* were co-transformed into the AH109 yeast strain. Co-transformed yeast clones were grown on a synthetic dropout medium minus Leu and Trp or Trp, Leu, and His with or without 3-AT(Sigma-Aldrich). The primers used to amplify promoters are listed in **Supplementary Table S1**.

### 2.6 Electrophoretic mobility shift assay

The coding sequence of *OsSPL3* was amplified by PCR and cloned into the pET28A vector, which has an N-terminal 6×His tag. The recombinant His-*OsSPL3* plasmid was transformed into *Escherichia coli* strain BL21 (DE3) and purified with a nickel-nitrilotriacetic acid (Ni-NTA) agarose. According to the manufacturer’s instructions, the electrophoretic mobility shift assay was performed using a LightShift chemiluminescent EMSA kit (Thermo Scientific, Rockford, USA). Probes containing the GTAC motifs derived from the *OsD10* promoter were labeled with 5′ FAM (fluorescein isothiocyanate) fluorescent dye were synthesized. Two complementary oligonucleotides were mixed in a water bath at 95°C for 5 min and cooled to room temperature for annealing to obtain double-stranded probes. The His-OsSPL3 purified protein and probe were incubated with EMSA binding buffer (125 mM Tris-HCl 8.0, 750 mM NaCl, 25 mM MgCl_2_, 100 mg mL^-1^ BSA, 50% Glycerol, 5 mM DTT, 125 ng μL^-1^ Salmon sperm DNA). The mixture was incubated at 25°C for 20 min and separated on 6% polyacrylamide gels in 0.5×TG buffer at 90 V for 90 min. The glass was dried and scanned in an automatic chemiluminescence system. Relevant primer and probe sequences are given in **Supplementary Table S1**.

### 2.7 Dual-luciferase transcriptional activity assay

The *OsD10* promoters were amplified and cloned into the modified pH2GW7 vector (with P35Smini promoter) containing the firefly luciferase (LUC) gene and the Renilla luciferase gene (LUC) as reporters. The *OsSPL3* full-length cDNA was cloned into the pEAQHT-DEST2 vector as an effector. The plasmids were transferred into rice protoplast. The luciferase activities were measured with the Dual-luciferase reporter assay system (Promega, Madison, WI, USA) according to the manufacturer’s instructions. Three independent transformations for each sample were carried out, and the relative reporter gene expression levels were expressed as the ratio of firefly LUC to Renilla luciferase (LUC/REN).

### 2.8 RNA extraction and qRT–PCR

The total RNA in rice tissues is extracted using the TRIzol reagent kit (Vazyme, Nanjing, China). The first-strand cDNA was synthesized from 3 μg total RNA using EasyScript One-Step gDNA Removal and cDNA Synthesis SuperMix Kit (TransGen, Beijing, China). qRT-PCR was performed using the 2×SYBR Green qPCR Mix (SparkJade, Jinan, China), and detection was performed using the Quantstudio™ 7 Flex Real-time PCR system (Applied Biosystems, Carlsbad, CA, USA). The rice Ubiquitin gene (OsUBQ) was used as an internal reference to normalize gene expression. The primer sequences for qRT–PCR are listed in **Supplementary Table S1**.

### 2.9 *Orobanche* germination bioassay

The stimulatory activity of root exudates on *Orobanche* germination was evaluated using a germination bioassay with *Orobanche cumana Wallr.*, as described in previous studies (Cardoso et al., 2014). Approximately 100 preconditioned *Orobanche* seeds were placed on a 9-mm diameter glass fiber filter paper disk. The disk was then exposed to 50 μL column-purified root exudates, subsequent to acetone evaporation. After incubation in darkness at 30°C for 48 hours, the germination rate was determined. Each biological five included three discs, and five replicates were conducted in total.

### 2.10 Statistical data analysis

Metabolic data were subject to normalization processing. The metabolites contents were normalized through divided the relative signal strengths of the metabolites by the strength of the internal standard (0.1 mg/L lidocaine) and then log2 transforming them for further normalization to improve the normality. *P* values were determined by two-sided unpaired t-test, compared with wild type.

## 3 Results

### 3.1 Metabolic profiling analyses of *d10* mutants and ZH11 polished rice

**Figure 1:**
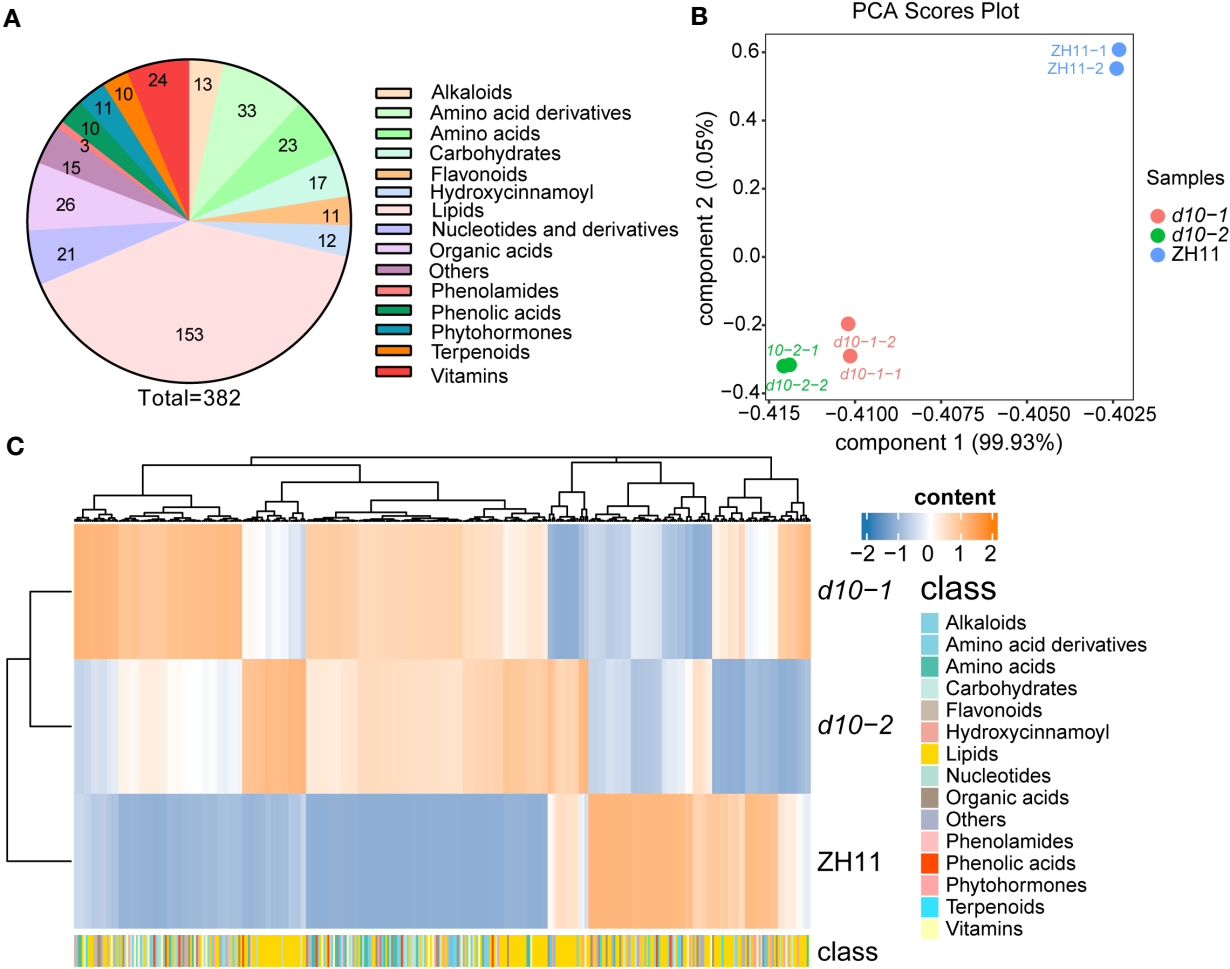
Metabolic profile analysis of polished rice in *d10* mutants. **(A)** Statistical analysis of various types of metabolites detected in this study. The numbers on the pie chart represent the amount of metabolites. **(B)** Principal component analysis (PCA) of the metabolites in *d10* and ZH11 polished rice. PC1 and PC2 refer to the first and second principal components, respectively. **(C)** Heatmap based on the metabolome data of *d10* and ZH11 polished rice. Heat map visualized with the average content of amino acids after normalization in two biological replicates. The yellow and blue colors indicate a high or low relative abundance, respectively. Each rice genotype is visualized in a single row, and a single column represents each metabolite. The bottom annotation with different colors represents a different class to which the corresponding metabolite belongs.

To obtain a comprehensive understanding of the impact of SL biosynthetic genes on the metabolic diversity of polished rice, a widely-targeted metabolomics approach using HPLC-ESI-MS/MS was employed (Chen et al., 2013). The study included the wild type (WT) line ZH11 and two allelic mutant of *OsD10*, characterized by dwarfism and increased tillering (**Figure S1**). A total of 382 metabolites were detected in polished rice (**Supplementary Table S2** ), including 153 lipids, 56 amino acids and their derivatives, 24 vitamins, 17 carbohydrates, 21 nucleotides and their derivatives, and 111 other secondary metabolites (**Figure 1A**).

Principal component analysis (PCA) was performed to explore the variability of the detected metabolites. Components 1 and 2 explained 99.93% and 0.05% of the variability, respectively (**Figure 1B**). The PCA score plots revealed distinct clustering of ZH11 and two *d10* mutants into separate groups. Moreover, the separation between ZH11 and the *d10* mutants was more pronounced than between the *d10* mutants (**Figure 1B**). Hierarchical cluster analysis based on the metabolome data further confirmed the metabolic differences between the *d10* mutants and ZH11 (**Figure 1C**). These findings suggest a potential role for *OsD10* in the metabolic reprogramming of polished rice.

To gain deeper insights into the metabolic divergence between *d10* mutants and ZH11, differentially accumulated metabolites (DAMs) with fold changes greater than 1.5 (*p*-value < 0.05) were identified. Approximately 36.1% of the 382 metabolites were classified as DAMs (**Figure 1C**, **Supplementary Table S2**). Among these, 120 up-DAMs exhibited higher levels, while 18 down-DAMs showed lower levels in the *d10* mutants than in ZH11. Further analysis revealed that the up-DAMs were predominantly composed of amino acids and their derivatives (41), lipids (15), vitamins (8), carbohydrates (8), nucleotides and their derivatives (12), organic acids and their derivatives (8), and phenolic acids (5). Notably, amino acids and their derivatives accounted for the highest proportion of up-DAMs, comprising 34.2% (**Table S2**).

### 3.2 Comparative analysis of amino acids accumulation pattern in *d10* mutants and ZH11 polished rice

**Figure 2:**
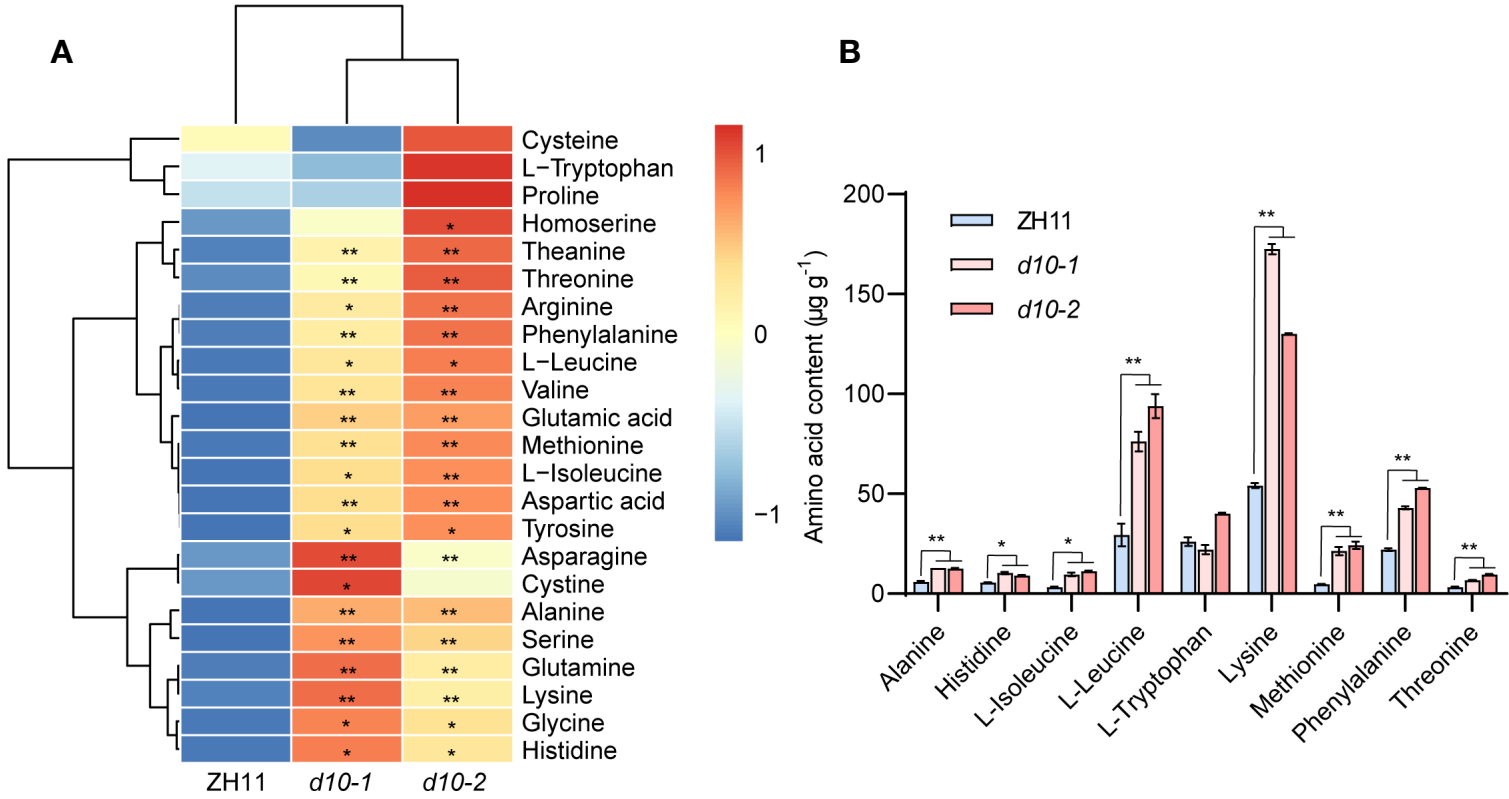
Metabolic profiling of amino acids in *d10* mutant polished rice. **(A)** Heat map visualized with the average content of amino acids after normalization in two biological replicates. The red and blue colors indicate a high and low relative abundance, respectively. **(B)** Nine essential amino acids content in *d10* mutants and ZH11 polished rice. *P* values were determined by two-sided unpaired *t*-test, compared with ZH11. Asterisks indicate significant differences (*, *p* < 0.05; **, *p* < 0.01).

Given the significance of rice as a vital source of amino acids for human nutrition (Shi et al., 2023), we comprehensively analyzed amino acid metabolites in both *d10* mutants and ZH11 polished rice. Among the 23 amino acids detected, except for cysteine, proline, and tryptophan, the remaining 20 amino acids significantly increased in the *d10* mutants, with glutamic acid and glutamine showing the highest fold changes, surpassing 5-fold (**Figure 2A**, **Supplementary Table S3**). Furthermore, when compared with ZH11, the *d10* mutants’ polished rice exhibited elevated levels of 7 essential amino acids (valine, leucine, isoleucine, tryptophan, threonine, lysine, and methionine) in the *d10* mutants’ polished rice were all increased by more than 0.5 times (**Figure 2B**, **Supplementary Table S3**). These findings strongly suggest that the loss-of-function mutation of *OsD10* promotes the accumulation of amino acids in polished rice.

### 3.3 Analysis of vitamins metabolic diversity between *d10* and ZH11 polished rice

**Figure 3:**
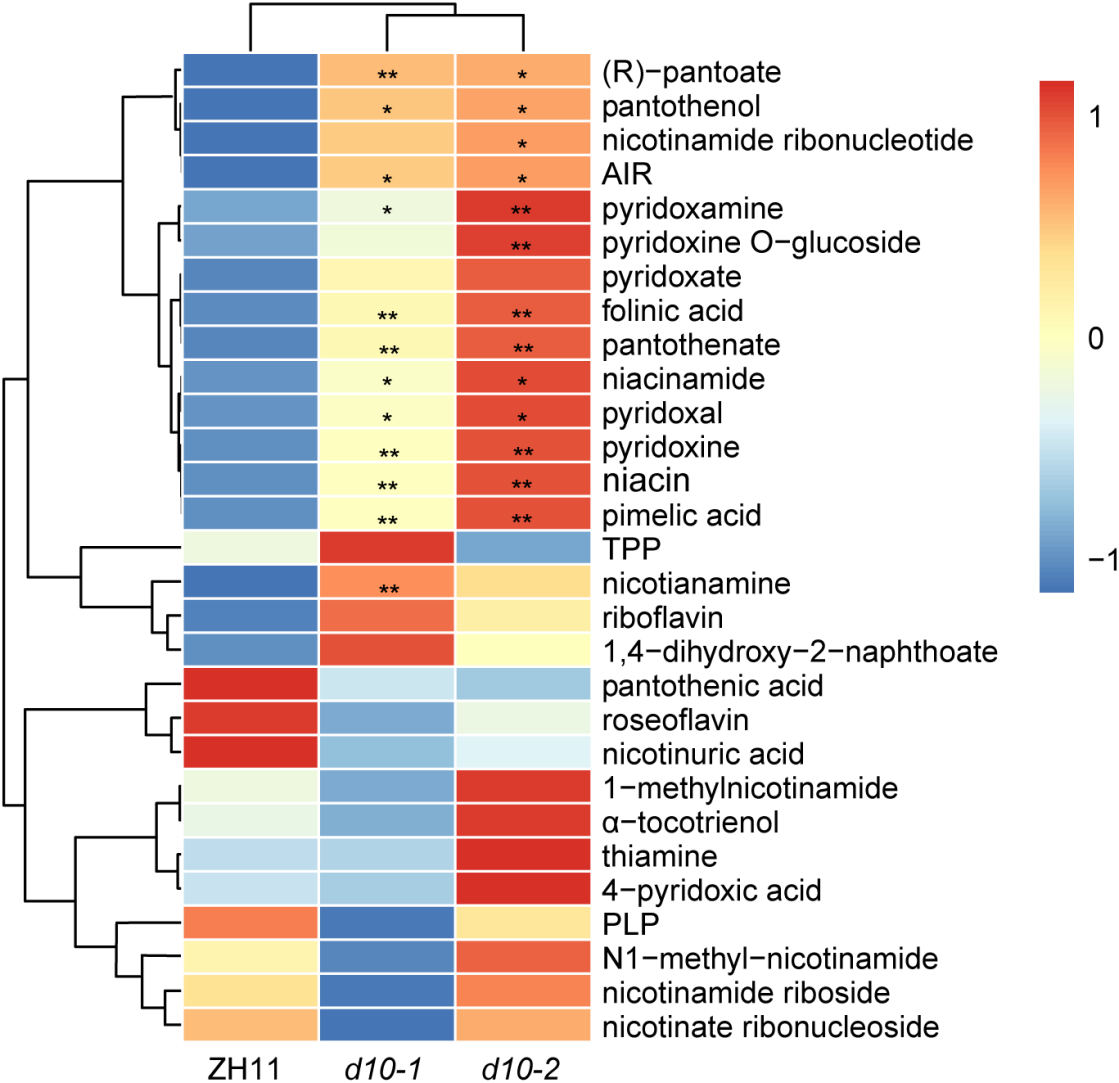
Metabolic profiling of vitamins in *d10* polished rice. Heat map visualized with the average relative content of vitamins after normalization in two biological replicates. The red and blue colors indicate a high and low relative abundance, respectively. *P* values were determined by two-sided unpaired *t*-test, compared with ZH11. Asterisks indicate significant differences (*, *p* < 0.05; **, *p* < 0.01).

Considering the essential role of vitamins in maintaining human health, we conducted a comprehensive analysis to investigate the influence of *OsD10* on vitamins accumulation. Compared with ZH11, the *d10* mutants exhibited significant increases in the levels of multiple vitamins (**Figure 3**). Specifically, the content of niacinamide (VB3), niacin (VB3), nicotinamide (VB3), (R)-thiazoline (VB3), (R)-pantothenic acid (VB5), panthenol (VB5), pantothenic acid (VB5), pyridineamine (VB6), vitamin B6 (VB6), pyridoxal (VB6), and folic acid (VB9) all displayed more than a 0.5-fold increase in *d10* mutants. Notably, the content of (R)-pantoate exhibited the highest increase, exceeding sevenfold. Conversely, the loss of *OsD10* function also decreased the accumulation of certain vitamins, such as roseoflavin (**Figure 3**, **Supplementary Table** **S4**). These findings suggest that *OsD10* is involved in the accumulation of multiple vitamins in polished rice.

### 3.4 Identification of OsSPL3 as a *OsD10* promoter-binding protein

**Figure 4:**
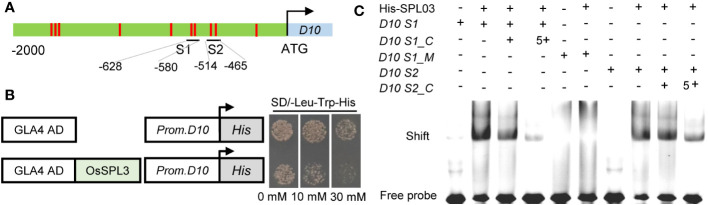
OsSPL3 directly binds to *OsD10* promoter. **(A)** Schematic of the *OsD10* promoter showing the OsSPL3 binding motif GTAC in red. S1 and S2 represent promoter fragments, each containing 2 GTAC motifs. **(B)** Yeast one‐hybrid assay results demonstrating the interaction between OsSPL3 and the *OsD10* promoter. The transformed yeast cells were grown on SD-Leu/-Trp/-His medium containing 0, 10, and 30 mM 3-AT, respectively. The *OsD10* promoter sequence used in the yeast one‐hybrid assay was a 189 bp fragment containing S1 and S2. **(C)** Electrophoretic mobility shift assays showed that OsSPL3 binds to the *OsD10* promoter via the GTAC motif. *D10 S1_C* and *D10 S2_C* represent competitive probes of *D10 S1* and *D10 S2*, respectively. In *D10 S1_ M*, motif GTAC was mutant to AGAC.

To identify potential regulatory transcription factors of *OsD10*, we performed a yeast one-hybrid (Y1H) screening using a *OsD10* promoter fragment. Among 12 obtained prey clones, five were identical to the transcription factor *SQUAMOSA promoter-binding PROTEIN-LIKE3* (*OsSPL3*), which has been reported to bind the GTAC motif (Shao et al., 2019). Through a cis-element analysis of the *OsD10* promoter, we identified 20 GTAC motifs distributed in 10 sites of *OsD10* promoter (**Figure 4A**).

To validate the interaction between OsSPL3 and *OsD10* promoter, we performed a Y1H assay using the truncated promoter of *OsD10* containing GTAC motifs. Yeast cells co-transformed with OsSPL3-AD and *OsD10* promoters exhibited robust growth in the presence of 30 mM 3-AT, whereas yeast cells containing empty-AD and *OsD10* promoter showed significant inhibition (**Figure 4B**). These results strongly support the interaction between OsSPL3 and the *OsD10* promoter.

We further employed electrophoretic mobility shift assays (EMSA) to demonstrate the direct binding of OsSPL3 to the *OsD10* promoter via the GTAC motif. Our data showed that OsSPL3 could bind to probe S1 and probe S2 (**Figure 4C**), and the binding affinity was substantially reduced in the presence of a competitive probe. Moreover, no binding band was observed when the GTAC motifs in probe *OsD10 S1* were mutated to the GAAC motif in probe *OsD10 S1_M*. These results prove that OsSPL3 directly binds to the *OsD10* promoter via the GTAC motifs.

### 3.5 OsSPL3 inhibits *OsD10* gene expression, thereby affecting the architecture of rice plants

**Figure 5:**
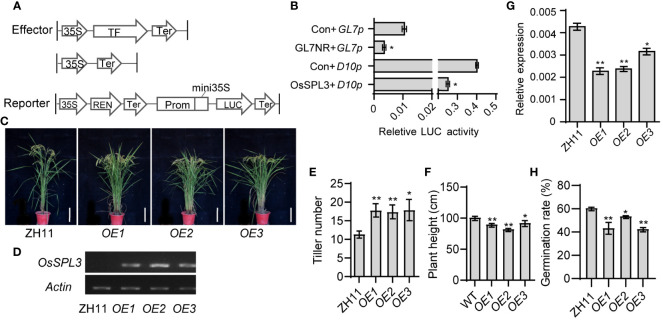
OsSPL3 inhibits *OsD10* transcription and regulates plant architecture of rice. **(A, B)** Expression activity assay of *OsD10* driven by OsSPL3 in rice protoplasts. Relative luciferase activity was monitored in rice protoplasts co-transfected with effector and reporter constructs. Con means empty effector construct. Effector GLN7NR and reporter *GL7p* as a positive control. Values are means ± SD (n = 3). **(C)** Performance of three independent *OsSPL3* overexpression (*OE1*, *OE2* and *OE3*) transgenic and ZH11 seedlings at heading stage. Scar bars, 20 cm. **(D)**The expression level of *OsSPL3* in its T3 overexpression lines (*OE-1*, *OE-2*, and *OE-3*) and ZH11 leaves. **(E, F)** Statistical analysis of tiller number **(E)** and plant height **(F)** in ZH11 and *OsSPL3.OEs* plants at the mature stage. Tiller number and plant height data are means ± SD (n = 15). **(G)** Expression levels of *OsD10* in leaves of *OsSPL3* overexpression plants with ZH11 background. RNA samples were collected from the second upper leaf of two-week-old plants. **(H)** The germination rate of *Orobanche* seeds treated with root extracts from *OsSPL3.OEs* and ZH11 plants, respectively. *P* values were determined by two-sided unpaired *t*-test, compared with ZH11. Asterisks indicate significant differences (*, *p* < 0.05; **, *p* < 0.01).

To assess the impact of the OsSPL3 protein on *OsD10* promoter activity, we conducted a dual luciferase (LUC) activity assay in rice protoplasts. Our results revealed that the relative LUC activity in protoplasts co-transfected with *OsSPL3* effector and *OsD10* reporter was significantly lower than that in the control group co-transfected with an empty vector effector and *OsD10* reporter (**Figure 5A, B**). The positive control, *GL7*, was reported to be repressed by GL7NR (Wang et al., 2015b), and our experimental results were consistent with this finding. These observations strongly suggest that OsSPL3 acts as a repressor on *OsD10*.

To further validate the regulation of OsSPL3 on *OsD10*, we generated transgenic plants overexpressing *OsSPL3* (*OsSPL3.OEs*) (**Figure 5C, D**). Remarkably, compared with ZH11, *OsSPL3.OEs* exhibited significant increases in tillering number while showing notable decreases in plant height (**Figure 5E, F**), resembling the phenotype of the *d10* mutants. The expression of *OsD10* was significantly reduced in the leaves of *OsSPL3.OEs* leaves than in ZH11 (**Figure 5G**), providing further evidence that OsSPL3 effectively inhibits *OsD10* transcription *in vivo*. Seed germination in *Orobanche* is stimulated by SL, and the germination rate serves as an indicator of SL levels (Cardoso et al., 2014; Xi et al., 2022). Thus, to investigate whether *OsSPL3* affects SL content, we performed an *Orobanche* germination bioassay. We treated the *Orobanche* seeds with extracts from the root of *OsSPL3.OEs* and ZH11. The germination rate induced by the extracts from *OsSPL3.OEs* was significantly lower than that induced by ZH11 extracts (**Figure 5H**). These results strongly suggest that *OsSPL3* likely suppresses *OsD10* expression, leading to inhibited SL accumulation and consequently thereby regulating rice plant architecture.

### 3.6 *OsSPL3* affects amino acids and vitamins accumulation in polished rice

**Figure 6:**
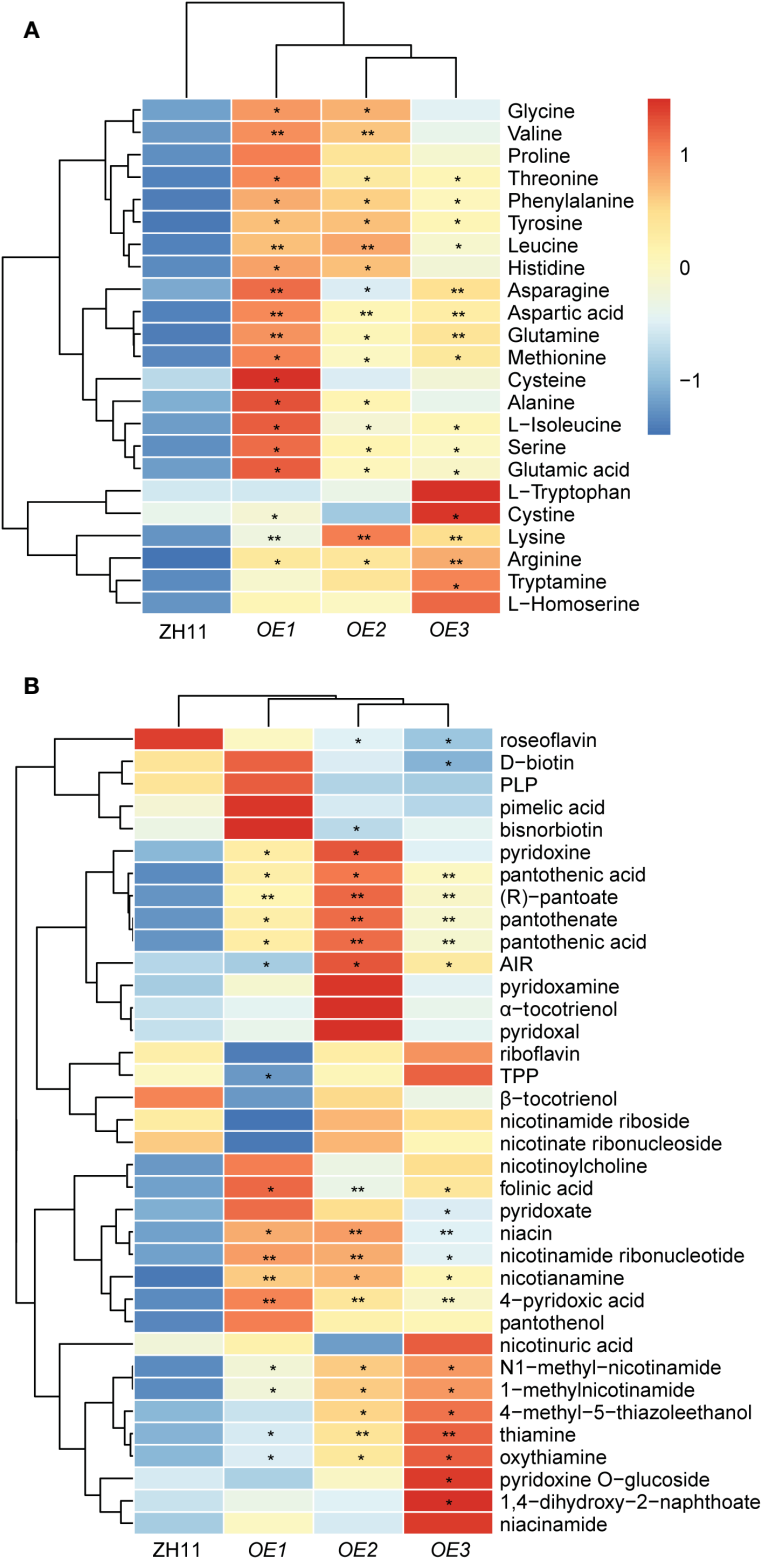
Accumulation pattern of amino acids and vitamins in polished rice of *OsSPL3* overexpression plants. **(A)** Heat map visualized with the average relative content of amino acids after normalization in two biological replicates. **(B)** Heat map visualized with the average relative content of vitamins after normalization in two biological replicates. The red and blue colors indicate a high and low relative abundance, respectively. *P* values were determined by two-sided unpaired *t*-test, compared with ZH11. Asterisks indicate significant differences (*, *p* < 0.05; **, *p* < 0.01).

Based on the observed relationship between *OsD10* and OsSPL3, we postulated that OsSPL3 may exert a regulatory role in amino acid accumulation in polished rice. To test this hypothesis, we comprehensively analyzed the metabolic profile in *OsSPL3.OEs* polished rice. Most amino acids in *OsSPL3.OEs* showed an increasing trend compared with ZH11 (**Figure 6A**, **Supplementary Table S5**). Notably, the contents of glycine, valine, threonine, phenylalanine, tyrosine, leucine, histidine, asparagine, aspartic acid, glutamine, methionine, alanine, isoleucine, serine, glutamic acid, lysine, and arginine were significantly higher in *OsSPL3.OEs* polished rice, with glutamine displaying the most pronounced increase, nearly threefold more elevated than in ZH11 (**Figure 6A**, **Supplementary Table S5**). These findings suggest that *OsSPL3* positively regulates the accumulation of most amino acids in polished rice.

Next, we assess the impact of OsSPL3 on vitamin accumulation. Our analysis revealed significant variations in the content of various B family vitamins between *OsSPL3.OEs* and ZH11. Specifically, compounds involved in the vitamin B1 synthesis pathway, including thiamine, thiamine oxide, 4-methyl-5-thiazole ethanol, and 5-amino-imidazole ribonucleotide (AIR), exhibited an increase of more than twofold in *OsSPL3.OE* polished rice compared to ZH11. Furthermore, notable increases in vitamin B3 levels were observed in *OsSPL3.OEs*, including niacin, niacinamide, N1-methylnicotinamide, and 1-methylnicotinamide. In addition, the content of vitamin B5, encompassing pantothenic acid, pantothenic acid, (R)-pantothenic acid, and pantothenic alcohol, also displayed a substantial increase of more than twofold in *OsSPL3.OEs*. Moreover, significantly elevated levels of vitamin B6 metabolites (4-pyridoxic acid, pyridoxamine, and pyridoxine) and folinic acid were observed in *OsSPL3.OEs* (**Figure 6B**; **Supplementary Table S6**). These findings provide compelling evidence that *OsSPL3* may play a broad positive regulatory role in the accumulation of various B vitamins.

## 4 Discussion

Despite extensive research efforts to unravel the biological function of SL and significant advances in comprehending their roles in regulating plant architecture (Yoneyama and Brewer, 2021) and stress resistance (Ha et al., 2014), the exact regulatory mechanisms of SL biosynthesis genes in plant nutrient metabolism, as well as their transcriptional regulation, remain poorly understood. Through an analysis of metabolic profiles using HPLC-MS/MS in polished rice of *d10* mutants, we identified that *OsD10* governs the accumulation of diverse primary and secondary metabolites, encompassing amino acids, vitamins, sugars, lipids, flavonoids, and alkaloids. Moreover, we unveiled a new transcriptional regulatory role of *SPLs* in SL synthetic gene regulation, specifically through the direct control of *OsD10* by OsSPL3. Furthermore, we showed that *OsSPL3* significantly influences the nutritional metabolism of polished rice.

In our previous study, we conducted a comprehensive analysis of metabolic changes in rice leaves comparing SL mutants with ZH11 variety, utilizing a widely targeted metabolomics approach (Zhou et al., 2020; Liu et al., 2022). In this current study, we employed an enhanced widely targeted metabolomics strategy to detect and analyze 382 metabolites in *d10* and ZH11 polished rice samples. Notably, significant differences were observed between *d10* and ZH11 in terms of amino acids and their derivatives, vitamins and their derivatives, nucleotides and derivatives, organic acids and their derivatives, carbohydrates, lipids, alkaloids, flavonoids, phenolamides, and phenolic acids present in polished rice (**Figure 1C**, **Supplementary Table S2**). More specifically, in *d10* polished rice samples, we observed significant increases in 20 amino acids and most amino acid derivatives compared to ZH11 (**Figure 2A**, **Supplementary Table S3**). Moreover, in *d10* mutants, Vitamin B3 (niacinamide, niacin, and nicotianamine), vitamin B5 (pantothenol, (R)-pantoate, and pantothenate), vitamin B6 (pyridoxamine, pyridoxine, and pyridoxal), vitamin B9 (folinic acid), and vitamin E (α-Tocopherol) showed increases exceeding 1.5 times the levels observed in ZH11 (**Supplementary Table S4**). While we identified 153 lipids in polished rice samples, approximately 15% in *d10* mutants displayed increases or decreases exceeding 0.5 times compared with ZH11 (**Supplementary Table S2**). Furthermore, most of the nucleotides and their derivatives, organic acids and their derivatives, as well as carbohydrates, exhibited an overall increasing trend in *d10* mutants (**Supplementary Table S2**). The comprehensive analysis of the metabolic data indicates that SL is vital in regulating metabolism in polished rice.

The accumulation of SL is finely regulated. Nutrient deficiency signals stimulate the expression of SL biosynthesis genes, leading to increased production of SL in various plant species (Yoneyama et al., 2012; Haider et al., 2023). Moreover, several hormones, such as auxin, abscisic acid, cytokinin, and gibberellin, have been shown to regulate SL biosynthesis in plants (Xu et al., 2015; Ito et al., 2017; Barbier et al., 2021; Tian et al., 2022). In addition, zaxinone, a novel endogenous carotenoid-derived molecule, negatively regulates root SL synthesis in rice by down-regulating the transcription levels of *D27*, *D17*, *D10*, and *CYP711A2* (Wang et al., 2019; Ablazov et al., 2020). Understanding the regulatory mechanisms underlying SL biosynthesis has been a long-standing inquiry. Recently, it has been reported that transcription factors such as PHR2 directly upregulate the expression of rice strigolactone synthesis genes *D17* and *D10* and directly regulate the transcription factors NODULATION SIGNALING PATHWAY1 (NSP1) and NSP2, which regulate the expression of *D27* gene in *Medicago truncatula* and rice to increase the SL accumulation (Liu et al., 2011). Although OsSPL14 directly regulates SL signaling pathways (Song et al., 2017), whether SPLs directly regulate SL synthesis genes remains unknown. In this study, we identified OsSPL3 binding to the *OsD10* promoter through Y1H screening and further confirmed the direct binding of OsSPL3 to the *OsD10* promoter through EMSA experiments (**Figure 4C**). Furthermore, we performed dual luciferase activity assays in rice protoplasts and verified the inhibition of the *OsD10* promoter activity by OsSPL3 *in vitro* (**Figure 5B**). Moreover, by detecting the *OsD10* transcription level in *OsSPL3.OEs*, we found that OsSPL3 inhibits *OsD10* transcription *in vivo* (**Figure 5D**). Our work has identified the functional role of the first SPL member in the transcriptional regulation of SL biosynthetic genes.

Recent studies have highlighted the crucial regulatory role of SPLs in plant metabolism. Examples include the regulation of anthocyanin accumulation by SPLs (Gou et al., 2011; Li et al., 2020), as well as their involvement in the synthesis of auxin (Li et al., 2021a) and carotene (Gao et al., 2018). However, the impact of *SPLs* on plant nutrient metabolism remains ambiguous. Previous investigations have demonstrated that OsSPL3 inhibits crown root development while exerting effects on the heading stage, plant height, and panicle size of rice (Shao et al., 2019; Jiang et al., 2020). Our study revealed a significant increase in the accumulation of most amino acids in *OsSPL3.OEs* polished rice, with glutamine exhibiting the highest accumulation (**Figure 6A**). This pattern of amino acid accumulation was similar to that observed in *d10* (**Figure 2**). Furthermore, the levels of various vitamins including B3, B5, B6, and folinic acid demonstrated a similar increasing trend in *OsSPL3.OE* and *d10* polished rice. Notably, (R)-pantoate exhibited the highest increase among the transgenic materials (**Figure 3**, **6B**). Consequently, it is reasonable to hypothesize that OsSPL3 and *OsD10* orchestrate the regulation of nutritional metabolism in polished rice through a transcriptional cascade.

## 5 Conclusion

This study demonstrates the impact of *OsD10* on the nutritional metabolism of rice, revealing significant increases in amino acids and vitamins in *d10* polished rice. Furthermore, our findings show the direct repression of *OsD10* transcription by *OsSPL3*, resulting in the enhanced accumulation of amino acids and multivitamins in polished rice. These findings provide insights into the transcriptional regulatory mechanism through which SL regulates the nutrient metabolism of polished rice, thereby opening avenues for breeding rice varieties with enhanced nutritional content.

## Data availability statement

The original contributions presented in the study are included in the article/**Supplementary Material**. Further inquiries can be directed to the corresponding author.

## Author contributions

KL: Formal analysis, Funding acquisition, Investigation, Writing – original draft. YC: Data curation, Formal analysis, Investigation, Visualization, Writing – review & editing. CF: Funding acquisition, Project administration, Supervision, Writing ‐ original draft, Writing – review & editing.
