# Supplementary material for: OsDWARF10, transcriptionally repressed by OsSPL3, regulates the nutritional metabolism of polished rice
Source: Front Plant Sci. 2023 Dec 7;14:1322463. doi: 10.3389/fpls.2023.1322463 (PMC10733476; doi:10.3389/fpls.2023.1322463)
Supplement: Supplementary file 2 [file DataSheet_1.docx]

Supplementary Material

# Supplementary Figures


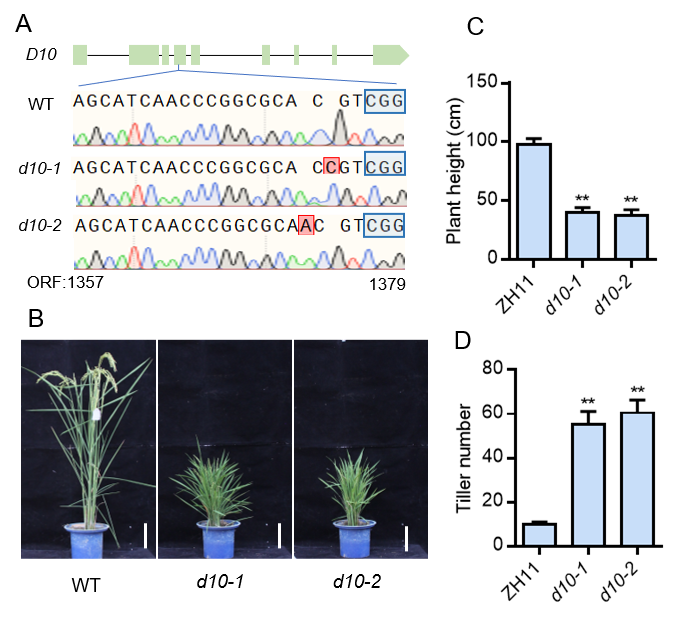


**Supplementary Figure 1.** Phenotype of *d10* mutant. **(A)** *D10* gene structure and *d10* mutant sequencing results. In the *D10* gene structure diagram, the green boxes represent coding regions with exons and the black line between boxes represents intron. The letter within red box in the sequence indicates the 1-bp insertion and the letters within blue boxes indicate the protospacer adjacent motif (PAM) recognition sequence of the Cas9 protein. **(B)** Phenotypes of WT and *d10* mutant plants at the heading stage. Scale bars, 15 cm. **(C-D)** Comparison of tiller number and plant height between WT and *d10* mutant plants. Data are means ± SEM (n = 12) and P-values are from two-sided Student’s t, **, P < 0.01.
